# Gender Gaps in Letter-Sound Knowledge Persist Across the First School Year

**DOI:** 10.3389/fpsyg.2018.00301

**Published:** 2018-03-08

**Authors:** Hermundur Sigmundsson, Adrian Dybfest Eriksen, Greta S. Ofteland, Monika Haga

**Affiliations:** ^1^Department of Psychology, Norwegian University of Science and Technology, Trondheim, Norway; ^2^Reykjavík University, Reykjavík, Iceland; ^3^Department of Neuromedicine and Movement Science, Norwegian University of Science and Technology, Trondheim, Norway

**Keywords:** children, gender differences, uppercase, lowercase, letter-name, letter-sound, longitudinal

## Abstract

Literacy is the cornerstone of a primary school education and enables the intellectual and social development of young children. Letter-sound knowledge has been identified as critical for developing proficiency in reading. This study explored the development of letter-sound knowledge in relation to gender during the first year of primary school. 485 Norwegian children aged 5–6 years completed assessment of letter-sound knowledge, i.e., uppercase letters- name; uppercase letter -sound; lowercase letters- name; lowercase letter-sound. The children were tested in the beginning, middle, and end of their first school year. The results revealed a clear gender difference in all four variables in favor of the girls which were relatively constant over time. Implications for understanding the role of gender and letter-sound knowledge for later reading performance are discussed.

## Introduction

Literacy is one of the main goals of primary education and is attained predominantly through reading, writing, speaking and listening (Rose, 2006). All of these domains facilitate children's intellectual, emotional and social development. A lack of basic reading skills at a young age is later associated with behavior problems and academic shortcomings (Adams, 1990; Elbaum et al., 2000; Tønnessen and Uppstad, 2015). At its most fundamental level, reading involves connecting vision to sound and attaching semantics toward these units of communication. Achieving a sufficient level of awareness and automaticity in reading requires a systematic knowledge of phonemes, i.e., knowing the sound of each letter in the alphabet (Hulme et al., 2012; Tønnessen and Uppstad, 2015).

### Importance of letter-sound knowledge for reading development

Literacy has a profound impact on the human brain (Dehaene, 2011). Recent brain imaging studies have identified specific locations in which symbols, such as letters, are processed. The importance of the visual word form area (VWFA) in the left lateral occipitotemporal sulcus is hypothesized to play a crucial role in human's processing of letters and words (Dehaene et al., 2002; Dehaene and Cohen, 2011). Dehaene and colleagues contend that the most influential factor for learning how to read is the attachment of meaning to symbols, of which letters and phonemes are the most crucial building block. In evolutionary term, reading is a relatively novel invention that relies on our finely tuned visual system to decode the meaning of symbols. This decoding process can be considered as a form of “neuronal recycling”: we use parts of the brain evolved for specific visual functioning in order to read. Connecting sound with these symbols, i.e., sublexical reading, is therefore of critical importance in order to decode whole words that compromise human language (Dehaene et al., 2010). As Tønnessen and Uppstad (2015) point out, the knowledge that words consist of a subset of sounds is an essential component of fruitful reading development. Furthermore, systematic phonics instruction seems to be essential in early reading skill acquirement (National Reading Panel, 2000; Ehri et al., 2001; Rose, 2006; Tønnessen and Uppstad, 2015). This includes learning all phonemes connected to each letter in the alphabet before learning to manipulate words, as this yields specific and advantageous cortical changes at an early stage in development (Dehaene et al., 2010; Dehaene, 2011). Hulme et al. (2012) found that letter-sound knowledge and phoneme awareness can both causally influence the development of early literacy skills. From a neurological perspective, the amount of systematic phonics teaching (i.e., learning letters one by one) in school seems to be the best predictor of reading comprehension in young children (Ehri et al., 2001). Similar results have been seen in adult readers, whereof grapheme- phoneme training yielded better results and more left lateralized specialization when learning to read texts with novel symbols (Yoncheva et al., 2015). Environmental factors seems not to be sufficient to explain the total variability in letter knowledge (Torppa et al., 2006), however, research concerning the cognitive basis of letter knowledge remain limited and largely unexplored (de Jong and Olson, 2004).

### Gender differences in reading

Matthews et al. (2009) highlighted the growing gender gap in academic achievement. Girls tend to progress more efficiently academically and to attain higher levels of education than boys (Birch and Gary, 1998; Silverman, 2003). Results from PISA, PIRLS, and the Norwegian National Tests confirm this view by indicating a gender gap in reading performance (Mullis et al., 2012; Stoet and Geary, 2013; OECD, 2014). According to recent PISA results, there is a significant difference in reading of 15-year-old Norwegian boys and girls (OECD, 2014). Although both genders are above the OECD average in reading, Norwegian girls outperform boys with an average score of 528 vs. 481 respectively. The average difference between boys and girls across the OECD is 38.

These, empirical observations raise questions as to how these gender differences arise. Maturation effects might explain some of the differences, for example, research has shown faster vocabulary growth in girls at an early age (Huttenlocher et al., 1991; McCune, 1992). Reznick and Goldfield (1989) found that vocabulary growth for children under 2 years old was faster for girls, and Hohm et al. (2007) found a significant difference between the genders at the age of 10 months in language in favor of the girls. Giedd et al. suggest that sex hormones might be an underlying factor (Giedd et al., 2012). The elevated levels of testosterone in the male fetal brain seem to slow the development of the left hemisphere, which could explain why boys are more likely to have reading difficulties (Geschwind and Galaburda, 1987; Tønnessen and Uppstad, 2015). Furthermore, boys seem to develop executive functions, such as processing speed and visuospatial working memory, at a slower rate than girls (Dekker et al., 2013; Stoet and Geary, 2013). Girls also seem to have deeper engagement and more motivation for reading (McKenna et al., 1995; Lynn and Mikk, 2009), as well as being more socially oriented; factors that could greatly affects language development from an early age (Halpern, 2012). Additionally, mothers tend to communicate verbally more frequently with girls than with boys (Halverson and Waldrop, 1970; Cherry and Lewis, 1978).

Based on the current evidence, development of letter-sound knowledge seems to have a multicausal explanation (Stoet and Geary, 2013). Regardless, the education system is the most prevalent environmental factor in the development of reading skills and, therefore, has a responsibility to create fruitful learning environments for both genders.

Letter-sound knowledge has been found to be crucial for later reading development (National Reading Panel, 2000; Piasta and Wagner, 2010; Hulme et al., 2012) and also shown to be the best predictor of children's future spelling and reading abilities (Scarborough, 1998; Hammill, 2004; Schatschneider et al., 2004). Research indicates a significant difference that favors girls, in letter-sound knowledge among Norwegian school-aged children (Sigmundsson et al., 2017a). However, research on gender differences in letter-sound knowledge is still limited (Dodd and Carr, 2003) and little is known on how these gender differences emerge in childhood. No studies have examined whether the gender gap in letter-sound knowledge changes longitudinally in a Norwegian speaking sample, this could be of importance since this language is considered to have a transparent orthography.

Broadening our understanding about this critical reading skill is important as it could influence and inform future teaching approaches. Therefore, the aim of this study was to examine how letter-sound knowledge develops in girls and boys during their first year of primary school. Based on the existing literature, it is reasonable to hypothesize that the gender-gap in letter-sound knowledge would tend to exist in the first year of school.

## Methods

### Study design and participants

A total of 485 children between five and six-years old were included in this study. The participants completed an assessment of letter-sound knowledge (Bokstavtesten) (Ofteland, 1992) at the beginning, middle, and end of the first year of primary school.

The children (*N* = 485, 224 girls and 261 boys), were selected from 28 schools in Norway (convenience sampling as the schools were invited to participate). The mean chronological age of the entire group at the start of the project was 6.1 years (*SD* = 0.3); the overall range was 5.67–6.67 years.

The schools varied in size and location (from urban to suburban), included pupils with different sociocultural- and economic backgrounds, and was representative of Norwegian 1st grade students. Exclusions criteria included; uncorrected visual deficit; behavioral, neurological or orthopedic condition; a history of learning difficulties or any other medical condition that could potentially interfere with the ability to carry out the tests.

### Measurements

#### Letter-sound knowledge

Letter-sound knowledge was assessed using the Norwegian version of the Letter-sound knowledge test (LSK test; “Bokstavtesten”; Ofteland, 1992). In the LSK test, participants are presented with the alphabet of printed letters, and verbally indicate how many of the uppercase- (e.g., “A, B, C,…”) and lowercase letter (e.g., “a, b, c,…”) they know the name and sound of. There are 29 letters in the Norwegian alphabet, which is based upon the Latin alphabet and is identical to the Danish alphabet.

The LSK test takes about 10 min to complete and consists of two sheets, one for the uppercase letters and one for lowercase letters.

The LSK test has proved to be a reliable and valid test of isolated word decoding proficiency (Ofteland, 1992). We estimated the convergent construct validity of the test battery by comparing the LSK ranking of the test scores of 20 Norwegian children (mean age 6.05 years, *SD* 0.28) with the rankings provided by the teacher of the same children. There was a moderate association between the rankings based on the teacher's evaluation and the rankings of test scores, with a Spearman rho correlation of 0.683.

The relative test-retest reliability of the test-battery was estimated using ICC (2, 1) (Shrout and Fleiss, 1979). The results were indicative of good reliability for individual test item scores, with ICCs between test and retest scores ranging from 0.985 to 0.992 (mean age 6.05 years, SD 0.28) in Norwegian children (*N* = 20) (Sigmundsson et al., 2017a).

#### Procedure

Full ethical review and approval were not required for this study in accordance with the national and institutional guidelines, however, the study was carried out in compliance with the recommendations of the Norwegian Centre for Research Data and the Declaration of Helsinki. Written informed documents were obtained from the parents of all participants prior to the study initiation. Identification numbers were used to maintain confidentiality.

The assessment took place in a quiet room during school hours and was conducted according to the LSK manual. All of the participants were tested individually by teachers trained in the test protocols.

Each test item was thoroughly explained before the participants started.

#### Data reduction and analysis

Statistical analyses were performed using SPSS Version 19 for Windows (SPSS Inc., Chicago, IL, USA). The occurrence of missing data was treated by listwise deletion. For the total score analysis and the analyses of lowercase letter-name and lowercase letter-sound the *N* was 411 (girls *n* = 186; boys *n* = 225). For the analyses of uppercase letter-name letter large name and uppercase letter-sound the *N* was 485 (girls *n* = 224; boys *n* = 261). Differences in letter-sound knowledge between girls and boys over time were assessed using the General Linear Model—repeated measure for the total scores of the four different measurements of letter-sound knowledge. Gender was used as the between-subjects factor, and time of testing was used as the repeated- measures factor. Statistical significance was set at *p* < 0.05. Because the assumption of sphericity was violated in the data sets analyzed by repeated measures, we applied the Greenhouse–Geisser correction.

## Results

Descriptive statistics of the scores for uppercase letters (name and sound), and lowercase letters (name and sound) for both genders over time are shown in Table 1. Higher scores indicate better performance (more knowledge of the letters and their sound).

**Table 1:** Descriptive statistics of score for amount of uppercase letters name, uppercase letters sound, lowercase letters name, and lowercase letters sound for 5–6 year old girls and boys.

	**Girls (*n* = 186)**	**Boys (*n* = 226)**
	**Mean (*SD*)**	**Mean (*SD*)**
**Uppercase letter-name** SEP	14.34 (10.10)	11.15 (8.64)
**Uppercase letter-name** JAN/FEB	21.71 (7.60)	18.73 (9.23)
**Uppercase letter-name** MAY/JUN	25.66 (5.45)	23.20 (7.79)
Uppercase letter-sound SEP	10.89 (9.95)	8.28 (8.39)
Uppercase letter-sound JAN/FEB	19.69 (8.39)	17.45 (9.43)
Uppercase letter-sound MAY/JUN	24.89 (5.84)	22.85 (7.71)
**Lowercase letter-name** SEP	10.35 (9.53)	7.31 (7.51)
**Lowercase letter-name** JAN/FEB	17.23 (9.08)	13.90 (9.28)
**Lowercase letter-name** MAY	22.58 (7.43)	19.84 (8.94)
Lowercase letter-sound SEP	8.62 (9.63)	5.86 (7.42)
Lowercase letter-sound JAN/FEB	16.27 (9.45)	13.12 (9.43)
Lowercase letter-sound MAY/JUN	22.00 (7.62)	19.40 (9.14)

*Higher scores indicate better performance (more knowledge of the letters and their sound)*.

**Figure 1:**
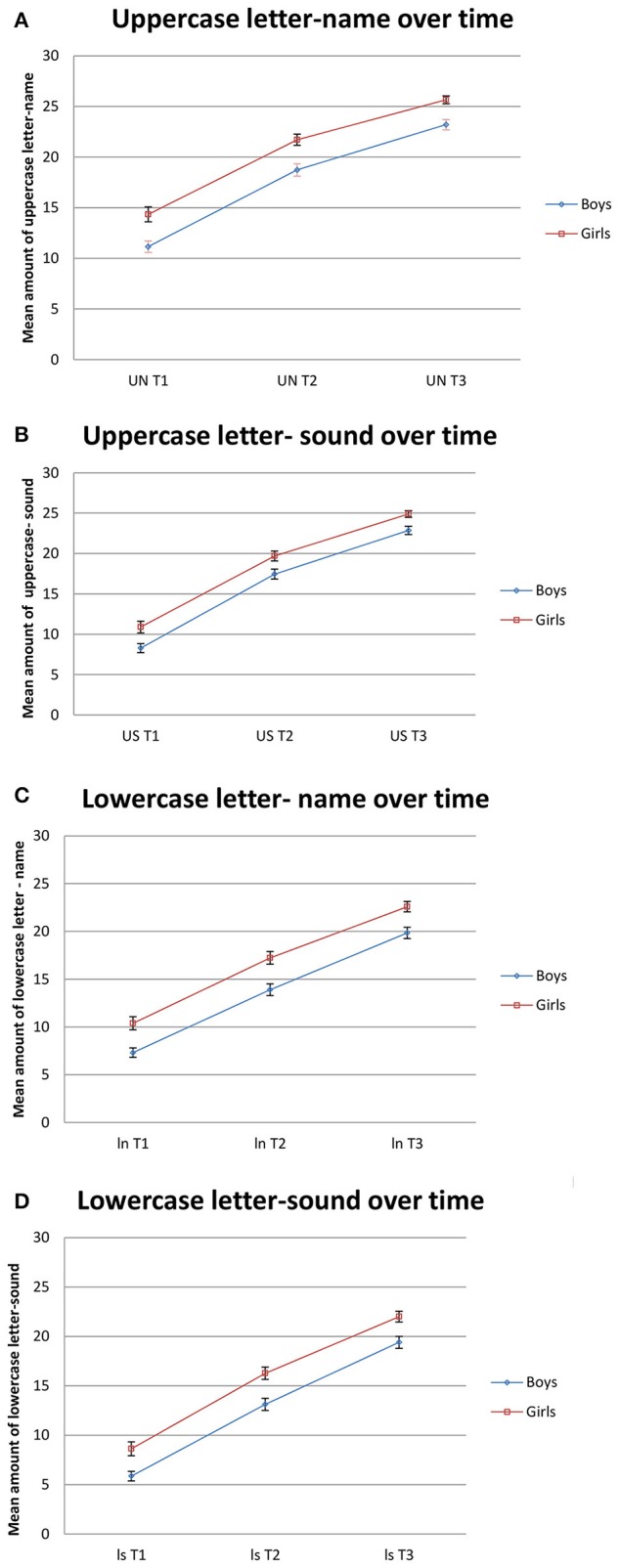
Letter–sound knowledge for girls and boys the first year at school. Mean score and standard error bars are presented. **(A)** Uppercase letter- name over time. **(B)** Uppercase letter-sound over time. **(C)** Lowercase letter- name over time. **(D)** Lowercase letter-sound over time. Time 1, September; Time 2, January/February. Time 3, May/June.

General linear model (repeated measures, mixed model analysis of variance) revealed significant main effect of gender [*F*_(1, 409)_ = 13,636, *p* < 0.001; partial η^2^ = 0.032]. Thus, there was an overall difference in letter-sound knowledge between girls and boys. A significant main effect was obtained for time [*F*_(1.553, 635)_ = 1,030, *p* < 0.001; partial η^2^ = 0.716], both groups scores significantly higher on the letter-sound knowledge measures after a period of 9 months. However, there was no significant time x gender interaction [*F*_(1.553, 635)_ = 0.456, ns; partial η^2^ = 0.001]. These results demonstrate that, over time, the development in letter-sound knowledge were similar for both girls and boys, but that the relative difference in performance between genders tended to persist (Figure 1).

The detailed results of the analysis of variance are reported below for each test item.

### Uppercase letter–name

A significant main effect was obtained for time [*F*_(1.535, 741)_ = 776, *p* < 0.001; partial η^2^ = 0.617].

A significant main effect also was obtained for gender [*F*_(1, 483)_ = 20.4, *p* < 0.001; partial η^2^ = 0.041]. There was no significant interaction effect [*F*_(1.535, 741)_ = 0.717, *p* = ns; partial η^2^ = 0.001].

### Uppercase letter-sound

A significant main effect was obtained for time [*F*_(1.577, 761)_ = 1,007, *p* < 0.001; partial η^2^ = 0.676].

A significant main effect also was obtained for gender [*F*_(1, 483)_ = 14.4, *p* < 0.001; partial η^2^ = 0.029]. There was no significant interaction effect [*F*_(1.577, 761)_ = 0.738, *p* = ns; partial η^2^ = 0.002].

### Lowercase letter-name

A significant main effect was obtained for time [*F*_(1.736, 709)_ = 779, *p* < 0.001; partial η^2^ = 0.656].

A significant main effect also was obtained for gender [*F*_(1, 409)_ = 15.1, *p* < 0.001; partial η^2^ = 0.036]. There was no significant interaction effect [*F*_(1, 736, 709)_ = 0.442, *p* = ns; partial η^2^ = 0.001].

### Lowercase letter-sound

A significant main effect was obtained for time [*F*_(1, 670, 683)_ = 798, *p* < 0.001; partial η^2^ = 0.661].

A significant main effect also was obtained for gender [*F*_(1, 409)_ = 13.1, *p* < 0.001; partial η^2^ = 0.031]. There was no significant interaction effect [*F*_(1, 670, 683)_ = 0.351, *p* = ns; partial η^2^ = 0.001].

## Discussion

The aim of this study was to examine how letter-sound knowledge develops in girls and boys during their first year of primary school. Four letter-sound knowledge factors were tracked over a 9-month period from September to June.

The main effect of gender revealed an overall difference in letter-sound knowledge between girls and boys, favoring girls on all the four factors. The boys performed worse than the girls in all four factors at all three-time points (Table 1 and Figure 1). These findings are in accordance with studies that report lower reading competency in 10-year-old (Mullis et al., 2003, 2007) and 14-year-old (OECD, 2014) boys.

A significant mean effect of time indicated that both groups scored significantly higher on the letter-sound knowledge test after a period of 9 months. There was no significant interaction effect, indicating that the relative differences in letter-sound knowledge outcomes between the genders were maintained over time (see Figure 1). The relative difference between uppercase letter- name/sounds and lowercase letter name/sounds followed the same pattern.

Firstly, the findings from our study indicate a gender gap in letter-sound knowledge that is already present when they first attend primary school, and secondly, that these differences tend to persist throughout their first year. Research has demonstrated the impact of letter-sound knowledge on future reading skills (Hulme et al., 2012; Torppa et al., 2018). The gender gap, already observed in 5-to-6-year-old children, accumulates and may be one of several factors that explain the gender differences found in PISA 2015. PISA 2015, which presents the performance of 15 year-old student's, reported that about 20% of students in OECD countries do not achieve baseline levels of reading proficiency. This percentage has remained stable since 2009. That study also revealed that girls outperform boys in all countries and economies (OECD, 2016). “*In 2012, 14% of boys and 9% of girls did not attain the PISA baseline level of proficiency in any of the three subjects measured in PISA – reading, mathematics and science*” (p. 13) (OECD, 2015).

Since dropping out is a dynamic developmental process with various influencing factors such as social equalities, early action should be taken at multiple levels. However, it is possible to argue that gender differences contribute to this complex phenomenon. Early childhood literacy is fundamental for almost all school subjects and the substantial amount of boys who fail to attain proficient reading skills represents a major challenge for education systems (Stanovich, 1986; Entwisle et al., 2007). Furthermore, it is easy to imagine how poor performance in these basic skills leads to decreased motivation for further practicing and learning, thereby perpetuating the vicious cycle of demotivation. The education system might also be exacerbating these gender differences if adapted teaching methods are not initiated.

Specific training and systematic practice (Kleim and Jones, 2008; Sigmundsson et al., 2013, 2017b) are required to effectively learn the letters of an alphabet and their phonetic usage. Therefore, one could reason that boys have less training/experience than girls at this age. In this respect, it seems wise to advocate for systematic and thorough learning of letters and their sounds as early as possible, at least by the first year of school. To close the gender gap, we need to firstly find out the level of each child in letter-sound knowledge and secondly provide each child with the right challenges for their intervention/training (Csikszentmihalyi, 1975, 2008). Thirdly we need to have good follow-up of children in relation to new challenges/training (Kleim and Jones, 2008). This is in line with Dehaene (2011) who argues that: “*Grapheme-phoneme correspondences must be systematically taught, one by one: the amount of such teaching is the best predictor of reading performance…*” (p. 26). Hulme et al. (2012) also supports the importance of letter-sound knowledge and phonemic skills and suggest that these factors should be directly taught to all children in the early stages of their academic careers. The arguments for using synthetic phonics are supported by a number of researches in both experimental studies and large scale assessments (National Reading Panel, 2000; Ehri et al., 2001; Levin et al., 2006; Rose, 2006).

## Author contributions

HS and MH: idea, analysis, and writing; AD: analysis and writing; GO: idea and writing.

### Conflict of interest statement

The authors declare that the research was conducted in the absence of any commercial or financial relationships that could be construed as a potential conflict of interest.
